# RNA-binding proteins as versatile metabolic regulators

**DOI:** 10.1038/s44324-024-00044-z

**Published:** 2025-01-13

**Authors:** Ellie Koletsou, Ina Huppertz

**Affiliations:** 1 Max Planck Institute for Biology of Ageing, Joseph-Stelzmann-Straße 9b, 50931 Cologne, Germany; 2 University of Cologne, Faculty of Mathematics and Natural Sciences, Cluster of Excellence Cellular Stress Responses in Aging-associated Diseases (CECAD), Joseph-Stelzmann-Straße 26, 50931 Cologne, Germany

**Keywords:** Metabolism, Metabolomics

## Abstract

Metabolic shifts are a hallmark of numerous biological processes, including the differentiation of stem cells along a specific lineage and the activation of diverse cell types, such as immune cells. This review examines the intricate energy metabolic alterations that occur in diverse biological settings, from embryonic development to adult tissue homoeostasis and disease states. In particular, we emphasise the regulatory function of RNA-binding proteins (RBPs) in coordinating these metabolic shifts and examine how they modulate key pathways, such as glycolysis and oxidative phosphorylation, to meet the dynamic cellular energy demands. This review highlights the various mechanisms by which RBPs regulate these changes, ranging from active involvement in the post-transcriptional regulation of metabolically relevant genes to alteration of an RBP’s function by specific RNAs, metabolites or growth factors. Finally, we consider how ageing and disease affect the function of RBPs and how RBPs can disrupt the delicate balance of metabolic regulation. Taken together, this review provides a comprehensive overview of the critical interplay between RBPs and metabolism and offers insights into potential therapeutic targets for regenerative medicine and age-related diseases.

## Energy metabolism

Energy metabolism represents a fundamental aspect of life encompassing the complex set of biochemical processes by which organisms convert nutrients into energy, which is then utilized to maintain vital functions, facilitate growth and enable adaptation to the surrounding environment. The concept of energy balance is central to these processes, whereby the intake, storage and expenditure of energy must be carefully regulated in order to maintain homoeostasis. The primary energy currency of the cell is adenosine triphosphate (ATP), which is produced both directly and indirectly through the conversion of carbohydrates, fats and proteins. Metabolism can be divided into two broad categories: catabolism, which involves the breakdown of molecules to produce energy, and anabolism, which encompasses the synthesis of essential macromolecules utilizing this energy. These two aspects of metabolism occur in a series of interrelated reactions that are essential for the development, growth and reproduction of an organism^1^. This review will focus on the catabolic arm of energy metabolism and the metabolic shifts that support specific cellular differentiation processes.

The central chemical pathways that constitute catabolism are glycolysis, the tricarboxylic acid cycle (also known as the Krebs cycle), oxidative phosphorylation (OXPHOS) and fatty acid metabolism. Glycolysis is an anaerobic process that occurs within the cytoplasm, resulting in the production of two molecules of pyruvate and two molecules of ATP from a single molecule of glucose^2^. Conversely under aerobic conditions, the pyruvate generated by glycolysis in the cytoplasm is transported to the mitochondria to fuel the tricarboxylic acid (TCA) cycle and OXPHOS, resulting in the production of ~36 molecules of adenosine triphosphate (ATP) per glucose molecule. Due to its efficiency, OXPHOS is typically the primary source of energy in terminally differentiated cells and under basal conditions. However, in certain circumstances, such as hypoxia or in specific cancer cells, there is a shift towards the glycolytic pathway, which is then used primarily for energy production. This process is referred to as the Warburg effect^3^. Furthermore, fatty acid metabolism plays a pivotal role in energy production, particularly during periods of elevated energy demand or nutrient deprivation, such as limited glucose availability^4^. Fatty acids are metabolized via β-oxidation in the mitochondria, resulting in the production of acetyl-CoA, which enters the TCA cycle and ultimately generates high levels of ATP^4^. Although there is a balance between these pathways, this equilibrium can be disrupted depending on the cellular conditions, oxygen availability and nutrient supply, enabling cells to adapt their energy production to meet their needs.

## Metabolic rewiring as stem cells exit pluripotency

Stem cell differentiation and specifically their exit from pluripotency provides an excellent illustration of a biological process that is typified by a substantial metabolic shift. This process entails a transition from a pluripotent state to more specialized cell types, accompanied by significant alterations in cellular metabolism. A pivotal aspect of this metabolic reprogramming is the shift from glycolysis, which is predominant in pluripotent stem cells (PSCs), to OXPHOS as cells embark on their differentiation^5–7^. It is noteworthy that glycolysis is not only prevalent in PSCs, but is also a requisite for the maintenance of their stemness. The elevated anaerobic glycolytic flux in PSCs is sustained by high expression levels of hexokinase II and the inactive form of pyruvate dehydrogenase. The latter blocks conversion from pyruvate to acetyl-CoA in mitochondria, which aligns with the hypoxic environment that mammalian embryos are initially exposed to during development^8,9^. A reduction in the expression of the mitochondrial pyruvate carrier (MPC) has also been demonstrated to be an effective and critical method for preserving stem cell proliferation^10^.

The metabolic switch that occurs during differentiation has significant implications for the epigenetic landscape of stem cells. Moussaieff et al.^11^ demonstrated that the reduction in glycolysis rates during differentiation is closely linked to a rapid decline in glycolysis-derived acetyl-CoA production. A reduction in acetyl-CoA levels has been demonstrated to have a detrimental impact on histone acetylation, resulting in its loss and, consequently, the dissolution of the open chromatin structure that is characteristic of PSCs^11–13^. Their research demonstrated that increased histone deacetylation is associated with the loss of pluripotency and occurs within the first hours of spontaneous differentiation. Furthermore, inhibitors acting downstream of acetyl-CoA were found to delay the exit from pluripotency.

In a related study, Carey et al.^14^ emphasized the importance of α-ketoglutarate (α-KG), a metabolite of the TCA cycle, in maintaining the pluripotency of naïve mouse embryonic stem cells (mESCs). In naïve stem cells, intracellular α-KG is derived from both glucose and glutamine catabolism and serves as an important cofactor for numerous epigenetic regulatory enzymes, including histone and DNA demethylases. The elevated ratio of α-KG to succinate promotes histone and DNA demethylation in naïve mESCs, thereby enhancing the expression of pluripotency-associated genes. External supplementation with cell-permeable α-KG has been demonstrated to support mESC self-renewal in vitro, whereas supplementation with cell-permeable succinate has been shown to induce differentiation^13,14^.

In addition to the metabolites generated by the TCA cycle, which connect mitochondrial metabolism to the transcriptional regulation of stem cells, significant alterations in mitochondrial morphology, number, and function are frequently observed during stem cell differentiation. The mitochondria of stem cells are typically fragmented with underdeveloped cristae and show a reduced production of reactive oxygen species (ROS). In contrast, terminally differentiated cells display a tubular and hyperfused mitochondrial network with increased ROS production^9,15–17^. It is noteworthy that Katajisto et al.^18^ demonstrated that in asymmetrically dividing human stem cells, young and old mitochondria are differentially distributed between daughter cells. The study revealed that daughter cells with fewer aged mitochondria were more likely to sustain stemness compared to their counterparts, indicating the potential role of mitochondrial age in stem cell fate determination^18^. Taken together, the metabolic reprogramming observed during stem cell differentiation involves a complex interplay between energy metabolism, epigenetic regulation, and mitochondrial dynamics.

## Metabolic rewiring during embryonic stem cell fate commitment

The metabolic transition from glycolysis to OXPHOS that occurs during PSC differentiation is not merely a consequence of differentiation; rather it plays an active role in regulating cell fate decisions. PSCs, derived from the inner cell mass of a blastocyst, are capable of differentiating into the three primary germ layers: mesoderm, endoderm, and ectoderm^19^. The metabolic shift observed during differentiation is intricately linked to the specification of these germ layers, influencing the emergence of distinct cellular lineages that will ultimately form all the tissues and organs of the body.

**Fig. 1:**
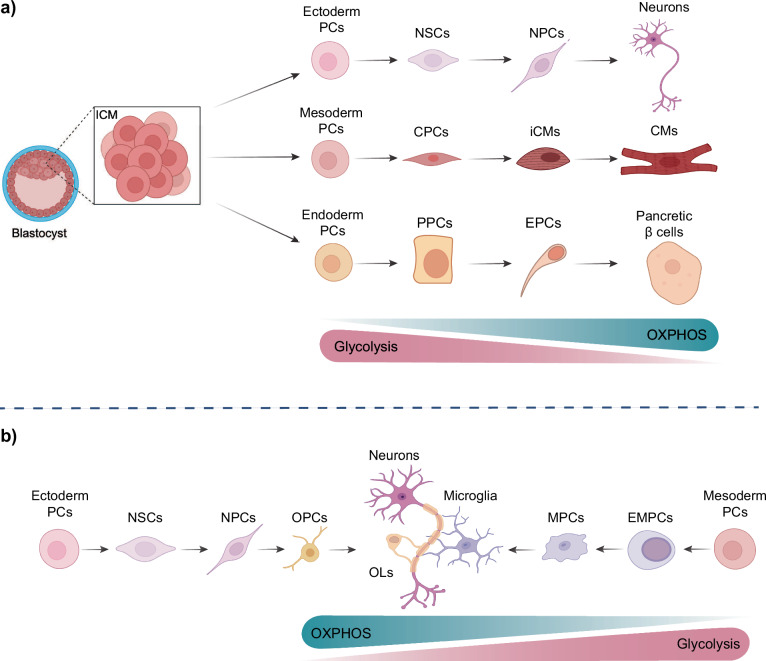
Metabolic rewiring underpinning embryonic stem cell differentiation. **a** The differentiation of pluripotent stem cells, derived from the blastocyst, to the three germ layers during embryonic development is characterised by a series of metabolic changes. During the differentiation towards a specific lineage, the majority of cells undergo a metabolic shift, from glycolysis to a more oxidative phosphorylation-based metabolism. In contrast to the mesodermal and endodermal precursor cells (PCs), the ectodermal PCs maintain their glycolytic activity for a longer period. **b** The differentiation process of the brain reveals distinct metabolic characteristics between two cell populations: oligodendrocytes and microglia. While oligodendrocyte precursor cells (OPCs) originate from neural progenitor cells (NPCs) and rely primarily on oxidative phosphorylation (OXPHOS) for ATP production, oligodendrocytes (OL) shift towards aerobic glycolysis and lipid synthesis during their continued differentiation. In contrast, microglia undergo a similar metabolic switch as neurons but are generated from mesodermal PCs. This illustration was created with BioRender. (NSCs: neural stem cells, NPCs: neural progenitor cells; CPCs: cardiac progenitor cells; iCMs: immature cardiomyocytes; CMs: cardiomyocytes; PPCs: pancreatic progenitor cells; EPCs (top): endocrine progenitor cells; OPCs: oligodendrocyte progenitor cells; OLs: oligodendrocytes; MPCs: microglial precursor cells; EMPCs (bottom): Erythroid myeloid progenitor cells).

To illustrate, as mesodermal cells differentiate into cardiomyocytes, they undergo a metabolic rewiring process that is specifically tailored to their successful cell-stage transition and maturation^20^ (Fig. 1a). While embryonic stem cells (ESCs) rely primarily on glucose and glutamine metabolism to generate energy, the differentiated cardiomyocytes utilize lactate and pyruvate derived from glycolysis for their TCA metabolism. This is reflected in the increased levels of TCA intermediate metabolites observed in human and mouse cardiomyocytes relative to undifferentiated PSCs^20–22^. Furthermore, culturing human and mouse PSC-derived cardiomyocytes in glucose-depleted media containing high levels of lactate results in selective elimination of undifferentiated PSCs and intermediate cells thereby yielding pure populations of cardiomyocytes^22^. The metabolic maturation of cardiomyocytes is characterized by an increase in fatty acid uptake and oxidation, which is accompanied by a rise in the proteins of the mitochondrial β-oxidation machinery, such as CPT1B^23^. At the cellular level, an increase in the number and mass of mitochondria is observed, accompanied by a fused and elongated mitochondrial phenotype containing complex cristae structures^21,23–25^. The process of mitochondrial fusion has been demonstrated to regulate cardiomyocyte differentiation via calcineurin and Notch signalling pathways. Following the depletion of the fusion proteins mitofusin 1 and 2, both the development of the mouse heart and the differentiation of mESCs into cardiomyocytes were arrested^26^.

The transition from a primarily glycolytic state to one that favours OXPHOS is also a feature of the endodermal lineage during development (Fig. 1a). A pertinent example is the differentiation of endodermal cells towards pancreatic insulin-producing β cells^27^. This metabolic shift is accompanied by increased mitochondrial biogenesis and enhanced expression of genes involved in the TCA cycle and the electron transport chain (ETC), which results in more efficient ATP production, thereby enabling the glucose-stimulated insulin secretion^28^. Concurrently, there is a pronounced upregulation in the expression of glucose transporters, particularly GLUT2, which mediates the enhanced glucose uptake indispensable for insulin secretion by these cells^29^. Additionally, the differentiating cells demonstrate augmented lipid metabolism through the increased expression of enzymes involved in fatty acid synthesis and β-oxidation^28^.

While the transition of mesodermal and endodermal cells from pluripotency is accompanied by a shift towards OXPHOS-mediated energy production, as described above, early ectodermal differentiation maintains its high glycolytic flux for a longer period^30^. Cells derived from the ectodermal lineage include, among others, neural stem cells (NSCs; Fig. 1a)^31^. This sustained glycolytic activity in ectodermal cells and NSCs is strongly correlated with and dependent on high levels of the MYC transcription factor family, which regulate the transcription of genes required for elevated glycolysis^30,32^. The MYC-dependent genes include the two enzymes, hexokinase 2 (HK2) and lactate dehydrogenase (LDH), whose downregulation, together with a shift in expression from pyruvate kinase muscle isoform 2 (PKM2) to PKM1, marks the transition from aerobic glycolysis in neural progenitor cells (NPCs) towards OXPHOS in neurons^33^. In addition to their differing glycolytic rates, NSCs possess elongated mitochondria, a feature that is not observed in the stem cells of other germ layers. As NSCs progress towards the NPC state, their mitochondria undergo fission, but return to the hyperfused state in terminally differentiated neurons.

NSCs also have the capacity to differentiate into cell types other than neurons, including oligodendrocytes. Oligodendrocytes are specialized brain-resident myelinating cells, and their precursors are the oligodendrocyte precursor cells (OPCs), which are derived from NPCs^34^. Despite originating from the same source, the metabolic switch that occurs during the differentiation of NSCs into oligodendrocytes and neurons represents a notable divergence in cellular energetics (Fig. 1b, left)^34^. Although both cell types undergo substantial metabolic reprogramming, the alterations are distinctive and adapted to their respective functions. As previously stated, neurons, which are highly energy-demanding cells, demonstrate a greater reliance on OXPHOS to meet their substantial ATP requirements for neurotransmission and maintenance of ion gradients^33^. In contrast, oligodendrocytes demonstrate a more pronounced shift towards aerobic glycolysis and lipid synthesis, in comparison to OPCs, which rely mainly on OXPHOS for ATP production^35,36^ (Fig. 1b, left). This metabolic adaptation in oligodendrocytes is essential for the severe membrane expansion required for myelin sheath formation^35^. Furthermore, oligodendrocytes upregulate pathways involved in cholesterol biosynthesis and fatty acid synthesis, which are crucial for myelin composition^37^. Another intriguing example of a brain-specific cell type is that of microglia. These cells undergo the same metabolic switch as NSCs during neurogenesis, relying primarily on OXPHOS for energy production under physiological conditions of inactivation^38^ (Fig. 1b, right side). However, in contrast to NSCs, these cells are derived from mesodermal cells, specifically from primitive macrophages, rather than from the ectodermal lineage^39^. During development, they migrate to the brain in a process orchestrated by NPCs^40^.

These divergent metabolic transitions illustrate the specialized nature of cellular differentiation and the complex relationship between metabolism and cell function in the central nervous system. An understanding of these intricate relationships provides valuable insights into the mechanisms governing stem cell fate and offers potential avenues for the manipulation of cellular differentiation in regenerative medicine and tissue engineering applications.

## Metabolic rewiring during adult stem cell and immune cell activation

Metabolic rewiring is not only a characteristic of cells during embryonic development, but also during the subsequent stages of adulthood. In adulthood, a variety of cell types undergo metabolic transitions in order to adapt to their physiological requirements. Specifically, neurogenesis is not only an early developmental process, but it can also occur in the adult brain of most mammalian species. In these species, NSCs are localized in restricted regions and exist in both a quiescent and an active state^41^. The quiescent state is defined as a reversible cell cycle arrest of cells stalled in the G_0_ or even in the G_2_ phase. These NSC pools have been observed in a range of species, including *Drosophila*^42^, mouse^43^ and human^44^. The precise regulation of quiescence and activation of stem cells is essential for the sustained maintenance of the stem cells pool, in addition to influencing the rates of neurogenesis^45,46^.

The transition of neural stem cells/neural progenitor cells (NSPCs) from a state of quiescence to one of activation is subject to stringent metabolic regulation. Quiescent NSPCs rely primarily on fatty acid oxidation (FAO) for energy production. Upon activation, they undergo a metabolic switch exhibiting decreased FAO and increased levels of de novo lipogenesis^47^. The reduction in FAO levels during the activation process enables the transition to a more active state, which is sufficient to promote the activation and proliferation of NSPCs. Conversely, the maintenance of high levels of FAO is a prerequisite for the support of the quiescent state^47,48^. Furthermore, in accordance with their increased dependence on FAO during quiescence, it has been demonstrated that NSPCs do not require glucose to sustain OXPHOS^49^.

Mitochondria play a significant role in the metabolic switch of adult NPSCs during the process of activation and proliferation. Upon activation, NSPCs shift to OXPHOS using the mitochondrial ETC for energy production. This transition is characterized by the increased expression of mitochondrial complexes, particularly in the transitional stage of fast proliferating progenitor cells^46,47,50^. Strikingly, it has been found that the mitochondrial pyruvate carrier (MPC), which facilitates the transport of pyruvate into mitochondria for utilization by the TCA cycle and the ETC, is expressed at high levels in quiescent NSPCs. These cells also demonstrate active mitochondrial metabolism^51^. These findings align with those of a previous study, which reported that the levels of ROS, generated by the ETC remain elevated in the quiescent NSPCs and decline in the activated state^52^. The active mitochondrial metabolism observed in these cells may be linked to the TCA metabolism, which is increased in quiescent NSPCs. These alterations in the metabolic landscape of the NPSCs is also corroborated at the level of gene expression, with single-cell RNA sequencing revealing a reduced expression of glycolytic genes and an increased expression of genes associated with OXPHOS, as previously mentioned, at early stages of activation^48,50^.

In addition to the proteins involved in mitochondrial complexes, which play a pivotal role in the process of NSPC activation, Wani and colleagues identified the function of a mitochondrial protease, YME1L, as a critical regulator of the rewiring of the mitochondrial proteome between the quiescent and active states of NSPCs. Specifically, YME1L activity is elevated in the quiescent state of NSPCs, facilitating the maintenance of their self-renewal capacity by regulating the abundance of numerous mitochondrial proteins. However, upon transitioning towards the active state, YME1L’s activity is diminished and a change in the mitochondrial proteomic landscape of the cells is observed^53^.

Another type of metabolic reprogramming is evident in immune cell activation, as exemplified by the activation of microglia cells^54^. For example, it has been demonstrated that exposure of microglia cells to amyloid-β (Aβ) plaques, which are pathological protein aggregates, results in a metabolic transition from OXPHOS to aerobic glycolysis^55^. This metabolic shift is associated with the mammalian target of rapamycin (mTOR)-hypoxia-inducible factor-1α (HIF-1α) pathway. Specifically, following exposure of microglia to Aβ plaques, it was observed that the AKT protein, which is an upstream activator of mTOR, underwent phosphorylation^55^. The phosphorylation of AKT promotes the phosphorylation of mTOR and ultimately the increased expression of HIF-1α, a key transcription factor regulating glycolysis^56^. Activation of microglia with other stimuli, such as lipopolysaccharide (LPS), can result in comparable metabolic rewiring, which occurs via the activation of the mTOR pathway. Inhibition of mTOR in microglia under these conditions has been observed to suppress the production of pro-inflammatory cytokines, which are pivotal contributors to immune and inflammatory responses^57^. Similarly, disparate immunological stimuli can prompt the activation of naïve T cells, which subsequently exit their quiescent state. This exit is accompanied by a shift from OXPHOS towards increased levels of glycolysis, lipogenesis and mitochondrial metabolism^58,59^.

In consideration of the collective evidence presented, it can be reasonably concluded that a multitude of factors are involved in the initiation of these metabolic transitions. However, the following discourse will focus on the emerging class of RNA-binding proteins, which offer a new avenue for the regulation of metabolism.

## RNA-binding proteins and their roles

RNA-binding proteins (RBPs) play crucial roles in regulating gene expression and RNA metabolism, particularly with regard to the lifecycle of mRNA, from synthesis to degradation. This topic has been the subject of extensive review by others^60^. In summary, these proteins bind to specific and diverse RNA sequences or structures, influencing the localization, stability, and translation of their target RNAs^60,61^. RBPs can act as positive or negative regulators of gene expression, depending on the context. This is achieved by modulating the accessibility of RNA to ribosomes, RNA-binding enzymes, or other regulatory factors^62^. To illustrate, some RBPs can enhance translation by recruiting ribosomes to specific mRNAs, whereas others impede translation by sequestering mRNAs into RNA granules or P-bodies^60^. Furthermore, RBPs can affect alternative splicing, RNA localization, and RNA decay, thereby modulating gene expression in response to cellular signals and environmental cues^60,61^. In addition to the regulation of RBPs on RNAs, it has been demonstrated that RNAs themselves can also actively control the function of RBPs upon binding to them. This type of regulation is referred to as *riboregulation*^63–65^. The dysregulation of RBPs has been linked to a number of diseases, including cancer, neurodegenerative disorders, and viral infections, which underscores the importance of these proteins in maintaining cellular homoeostasis. This review will focus on the significance of RBPs in the regulation of metabolic changes that cells undergo in different instances.

## Examples of RNA-binding proteins in metabolic regulation

### RNA-binding proteins as oxygen-responsive proteins

RBPs can affect metabolic pathways and the aforementioned metabolic transitions in a number of ways. One such mechanism has been elucidated by the recent discovery that there are specific clusters of RBPs which are responsive to oxygen availability, and enable the cells’ shift towards glycolysis. Ho et al.^66^ demonstrated that these “hypoxia-adaptive” RBPs, with HuR, PCBP1 and hnRNP A2/B1 being top candidates, enhance the translational efficiency of hypoxia-responsive proteins during oxygen deprivation, by promoting translational elongation. The proteins whose expression is regulated by these RBPs include glycolytic enzymes, such as enolase 1 (ENO1), aldolase A and lactate dehydrogenase A (LDHA). The underlying mechanism was found to rely on the association of these RBPs with the hypoxic protein synthesis machinery, which acts as a checkpoint for efficient translation and thereby facilitates the metabolic shift towards glycolysis and promotes cellular survival under hypoxic conditions^66^.

### Modulation of RNA-binding proteins by growth factors to adjust cellular metabolism

**Fig. 2:**
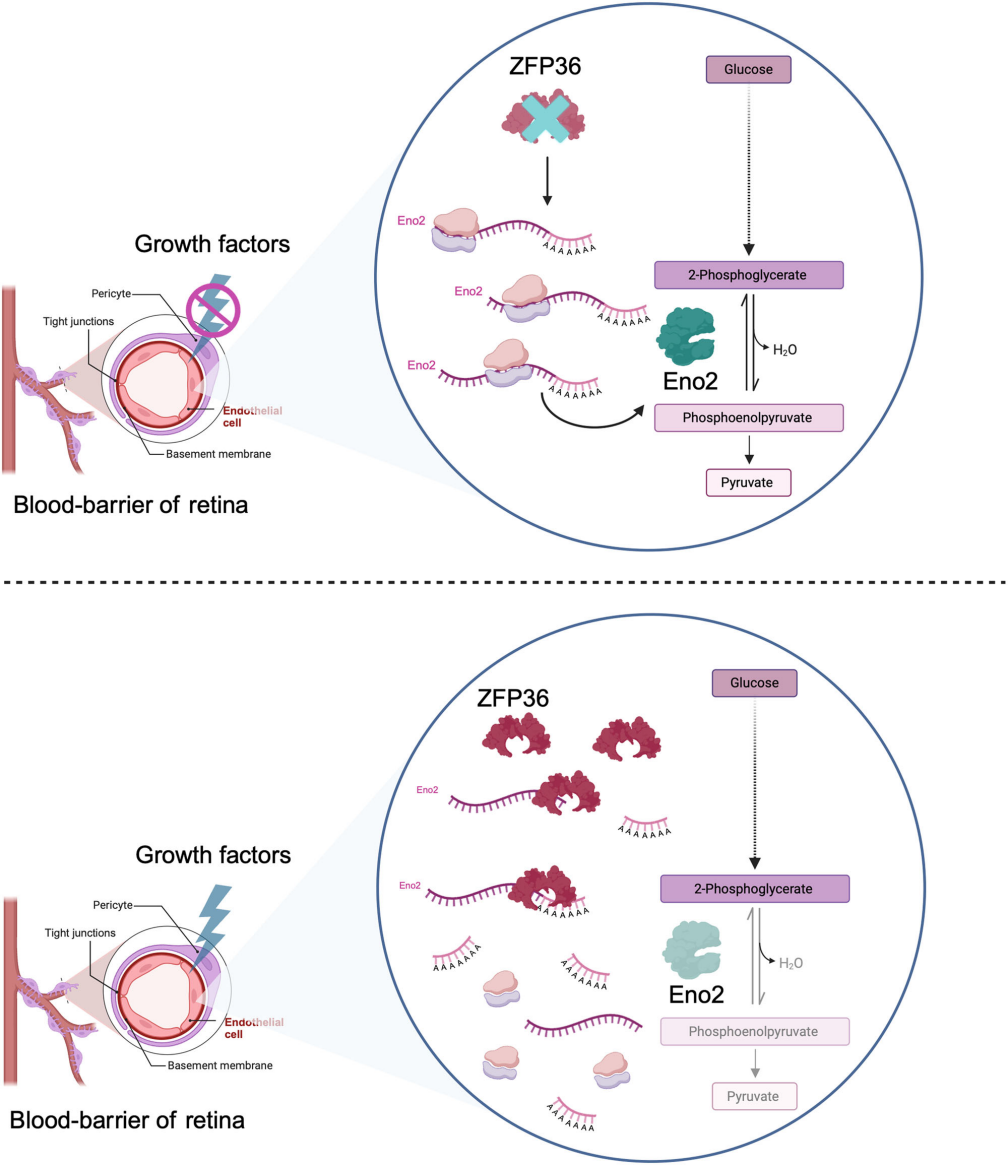
The RNA-binding protein ZFP36 restricts ENO2 mRNA expression to finetune the glycolytic activity in retinal neural cells. In the absence of growth factors, particularly VEGF, the RBP ZFP36 is not expressed in the endothelial cells of the retina. This results in the translation of the mRNA of ENO2, leading to increased levels of the glycolytic enzyme ENO2 and, consequently, the promotion of glycolysis. Conversely, the signalling of growth factors (i.e. VEGF) that enhance ZFP36 expression results in the decay of ENO2 mRNA through the deadenylation activity of ZFP36, consequently leading to decreased levels of ENO2 expression and thus a reduction in glycolysis, which is linked with retinal angiogenesis in vivo. The figure was created with BioRender.

Growth factors emerge as a further crucial building block linking RBPs to metabolic regulation. This connection is established through the modulation of metabolism-related gene transcription by factors such as MYC^30,32^. Intriguingly, Cicchetto and colleagues^67^ have identified an additional level of intricacy within this regulatory network. The researchers demonstrated that growth factors, such as insulin, VEGF, IGF-1, bFGF or PDGF-BB (depending on the cell type), can induce the expression of a specific RBP, namely ZFP36. This induction is performed via the MAPK/mTOR pathway. Upon activation, ZFP36 directly interacts with mRNAs encoding metabolic enzymes and nutrient transporters. Of particular interest is ZFP36’s interaction with the mRNA of the glycolytic enzyme enolase 2 (ENO2). This binding event results in the deadenylation of the ENO2 mRNA poly-A tail, which subsequently leads to its decay and a consequent reduction in protein expression. The resulting decrease in ENO2 protein levels manifests as diminished glycolytic activity, a phenomenon that is particularly pronounced in retinal neural cells where ENO2 is predominantly expressed^67^ (Fig. 2).

### RNA-binding proteins as regulators of mitochondria

One notable example of an RBP that regulates mitochondria is that of the Clustered Mitochondria Homologue (CLUH). CLUH is an evolutionarily conserved cytosolic RBP that plays a crucial role in regulating mitochondrial metabolism and biogenesis^68^. It has been demonstrated that CLUH specifically binds to nuclear-encoded mitochondrial mRNAs, promoting their translation and ensuring their stability^68^. A deficiency in this protein results in a significant remodelling of the mitochondrial proteome, with ~40% of mitochondrial proteins exhibiting reduced abundance in its absence^68^. The protein forms granular structures within the cytosol, and it is postulated that it coordinates the translation of mitochondrial proteins via mTORC1 signalling^69^. Although CLUH is not a primary component of the translation machinery, it interacts with it^70^ and plays a pivotal role in coordinating the expression of mitochondrial proteins involved in essential catabolic and energy-converting pathways. For example, it plays a role in the TCA cycle and β-oxidation^68^, particularly during conditions of nutrient deficiency such as the foetal-neonatal transition and starvation^69^. A reduction in mitochondrial proteins, which is associated with the loss of CLUH results in the loss of mitochondrial DNA (mtDNA) and abnormalities in the ETC and TCA cycle enzymes^68^.

Phenotypically, the expression of CLUH is increased during adipogenesis, which is mediated by cAMP/Creb signalling. In this context the protein is indispensable for the correct differentiation of adipocytes, mitochondrial respiration and the expression of brown adipocyte-specific genes^71^. Furthermore, CLUH is active in the growth cones of long neuronal axons, where it supports the local translation of mitochondrial proteins that are encoded from the nuclear genome^70^. Consequently, the maintenance of functional mitochondria in these distant compartments, particularly during the development of spinal motoneurons, requires CLUH^70^.

### RNA-binding proteins as regulators of mitophagy

Another intriguing aspect of the function of RBPs in the control of mitochondrial metabolism is their capacity to regulate mitophagy, particularly in neurons, which can exert an indirect influence on metabolic pathways. The PTEN-induced kinase 1 (PINK1) mRNA provides an interesting example of this mechanism. The association of PINK1 mRNA with the RBP synaptojanin 2 (SYNJ2), which in turn interacts with the mitochondrial outer membrane protein synaptojanin 2-binding protein (SYNJ2BP), enables the transport of PINK1 mRNA along with mitochondria. This process allows the PINK1 transcript to be available at distant locations, for example in dendrites, where it can be locally translated. This localized translation provides mitochondria with the otherwise short-lived PINK1 protein, which is essential for mitophagy. This mechanism is of particular importance in neurons, where mitochondria must travel long distances from the cell body^72^.

## RNA-binding proteins orchestrating metabolic transitions in stem cell fate

The complex relationship between RBPs and cellular metabolism represents a fascinating and intricate interplay, with a particularly striking manifestation observed in the context of stem cells. A number of RBPs are instrumental in the determination of cellular identity and function, particularly during the process of differentiation. One representative example is DDX21, an RNA helicase that plays a central role in epidermal tissue differentiation through a glucose-dependent mechanism. Briefly, this mechanism is driven by glucose, whose levels are increased during differentiation, and which binds directly to DDX21’s ATP-binding domain, inducing a conformational change. This confirmational alteration results in the dissociation of the DDX21 dimer inhibiting its helicase activity. This glucose-induced change prompts DDX21 to relocate from the nucleolus to the nucleoplasm, where it forms larger complexes with RNA splicing factors, thus regulating the splicing of genes that are crucial for epidermal differentiation^73^.

Another mechanism by which RBPs may influence the metabolic regulation of stem cells is through the action of RNA-binding transcription factors. For example, a study by Dvir and colleagues^74^ utilizing single-end enhanced crosslinking and immunoprecipitation (seCLIP-seq) demonstrated that the transcription factor STAT3, which is critical for pluripotency maintenance in mice^75^, can bind to multiple RNA molecules in human embryonic stem cells (hESCs). Notably, STAT3 binds to the long non-coding RNA (lncRNA) NORAD (non-coding RNA activated by DNA damage). However, this interaction does not affect NORAD itself, suggesting that the RNA may instead affect the function of STAT3. In addition to its role in pluripotency, STAT3 has been shown to have a significant impact on cellular metabolism. STAT3 has the ability to modulate amino acid utilisation, including processes such as glutamine uptake by promoting the upregulation of the neutral amino acid transporter gene SLC1A5, and the regulation of OXPHOS in leukaemia stem cells^76^. It also promotes the expression of genes involved in OXPHOS and mitochondrial respiration^77^, and accelerates the conversion of pyruvate to acetyl-coA^78^, thereby influencing ATP production and ROS levels. In some contexts, STAT3 can also favour aerobic glycolysis, a metabolic shift often observed in cancer cells^79^, making its regulatory role quite complex. The researchers further discovered that OCT4, a central transcription factor in pluripotency, also functions as an RBP in hESCs. This finding adds a new dimension to the role of OCT4 in pluripotency, suggesting that it may regulate gene expression not only through transcriptional control but also through direct RNA interactions^74^.

The RBP Lin28 represents an additional key factor influencing stem cell differentiation by interfering with metabolism. This is achieved by controlling the expression of microRNAs that target mRNAs involved in metabolism, thereby influencing energy balance and cellular growth during differentiation^80^. More specifically, Lin28 plays a crucial role in regulating the biogenesis of the Let-7 family of microRNAs. Lin28 inhibits the maturation of Let-7 from its precursor form, hence preventing the exertion of Let-7’s gene-silencing effects^81^. This interaction is of particular significance in the context of stem cell differentiation, given that Let-7 is known to promote differentiation and suppress self-renewal^82^. Consequently, Lin28 helps maintaining the pluripotency and self-renewal capabilities of stem cells by keeping Let-7 levels low.

A recent study has provided further evidence of a direct link between RNA binding and metabolism. The study revealed that the glycolytic enzyme ENO1 also acts as an RBP. Intriguingly, unlike conventional RBPs, ENO1 does not regulate its associated mRNAs. Instead, the mRNAs exert influence over ENO1’s activity (riboregulation). The riboregulation of ENO1 is necessary for the productive differentiation of ESCs. The mechanism entails the binding of specific RNA molecules to ENO1, which in turn inhibit its enzymatic activity. As ESCs undergo differentiation, the acetylation of ENO1 increases, enhancing its interaction with RNA and leading to a decrease in glycolytic activity. This reduction in glycolysis is essential for the differentiation of stem cells into various germ layers, indicating that ENO1’s riboregulation is a key factor in cellular fate determination^65^. It seems reasonable to propose that riboregulation may represent a more expansive mode of metabolic regulation, given that a considerable number of metabolic enzymes have been previously identified as RBPs in large-scale proteomic studies^63,83–86^, and other pivotal metabolic enzymes were found to be riboregulated, including SHMT1^87–89^.

## RNA-binding proteins in ageing and disease

The ageing process is a complex biological phenomenon defined by the progressive decline of physiological functions and increased vulnerability to disease and death. In 2013, López-Otín et al.^90^ put forth a conceptual framework comprising nine hallmarks of ageing: genomic instability, telomere attrition, epigenetic alterations, loss of proteostasis, deregulated nutrient sensing, mitochondrial dysfunction, cellular senescence, stem cell exhaustion, and altered intercellular communication^90^. Subsequently, they expanded their original set of hallmarks of ageing, proposing an updated framework that includes both primary and integrative hallmarks^91^. These interconnected hallmarks provide a framework for understanding the molecular and cellular mechanisms underlying the ageing process. In recent years, emerging evidence has highlighted the crucial role of RBPs in modulating various aspects of these hallmarks. RBPs, which regulate gene expression at the post-transcriptional level, have been implicated in diverse cellular processes, including mRNA stability, splicing, and translation. As such, their dysregulation can significantly impact the ageing process by influencing multiple hallmarks simultaneously. In the next sections we focus on how some of these hallmarks alter RBPs’ function or are altered by the abnormal function of RBPs, with a specific focus on metabolic derailment. In light of the extensive existing literature on the complex interplay between RBPs and metabolic alterations in the context of diabetes^92–94^, this review will not undertake a detailed examination of this subject within the scope of this review.

### Examples of RNA-binding proteins as metabolic regulators in cancer

One of the diseases that can manifest during the ageing process is cancer. Cancer can be broadly characterised by severe metabolic rewiring and is the result of a multitude of factors malfunctioning, including the accumulation of epigenetic alterations in the genome. Following transformation, some cancer cells appear to exhibit a metabolic profile analogous in many respects to that of stem cells. This is evidenced by their reliance on glycolysis for ATP production, even in the presence of high levels of oxygen that can efficiently support OXPHOS. This phenomenon is referred to as the “Warburg effect” and involves increased glucose uptake and fermentation of glucose to lactate, even with functioning mitochondria^95^. In addition to providing sufficient ATP, this metabolic shift confers several advantages on cancer cells, including the generation of biomass precursors, such as nucleotides, amino acids, and lipids, which are essential for rapid cell division^96^. Furthermore, the acidic by-products of glycolysis can create a favourable microenvironment for tumour growth and suppress the immune system’s response^97^. This metabolic rewiring enables the cells to meet the heightened energetic and biosynthetic demands associated with rapid proliferation and survival in the challenging tumour microenvironment. It is now established that RBPs also regulate metabolic processes in the context of cancer.

**Fig. 3:**
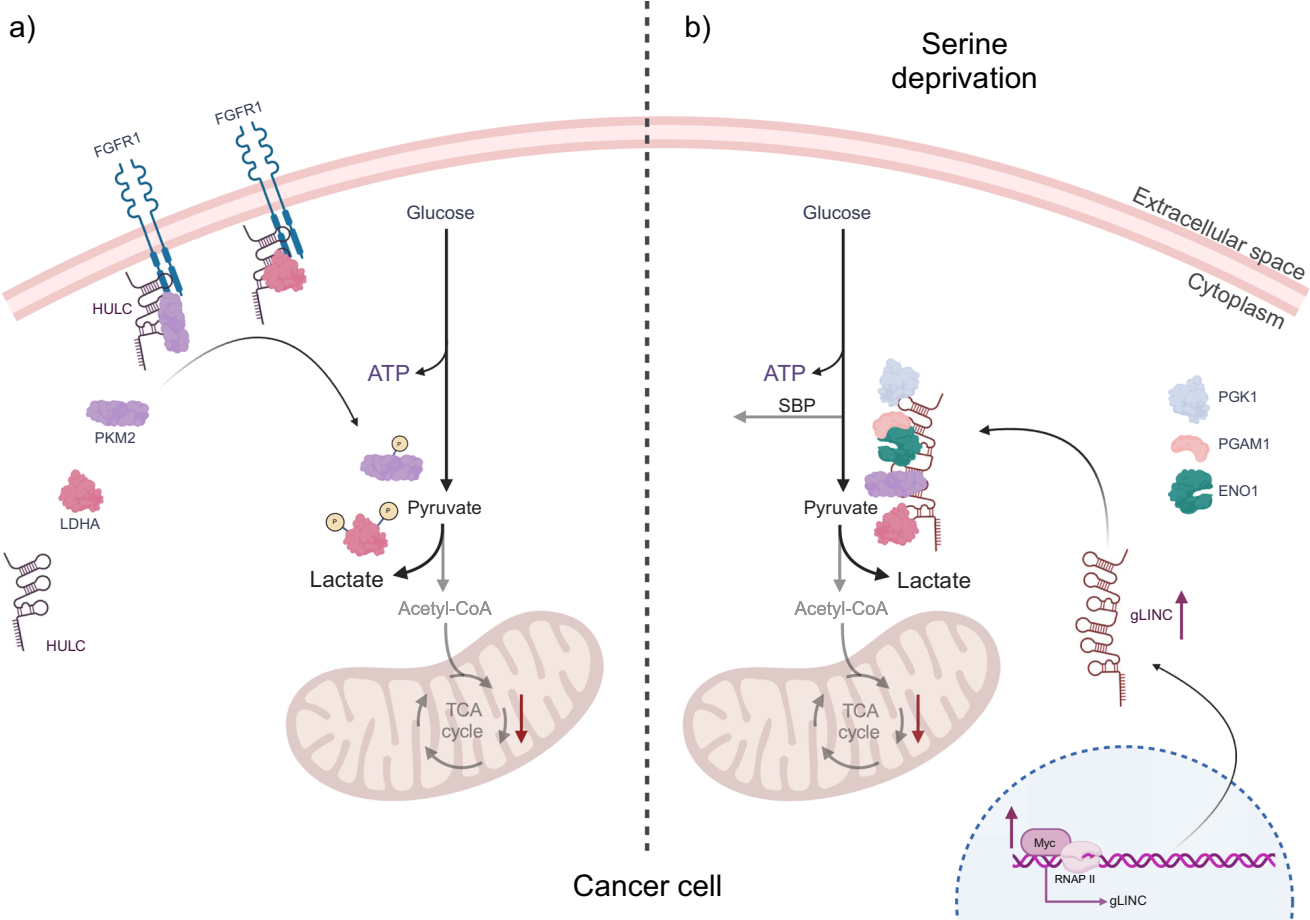
The function of two long non-coding RNAs (lncRNA) in the regulation of metabolic enzymes in cancer cells. **a** In cancer cells, the long non-coding RNA Highly Upregulated in Liver Cancer (lncRNA HULC) serves as a molecular scaffold for the glycolytic enzymes PKM2 and LDHA in conjunction with the receptor FGFR1. Binding of the glycolytic enzymes to FGFR1, mediated by HULC, results in their phosphorylation, thereby regulating the glycolytic pathway and ensuring efficient ATP production for cell survival. **b** In response to serine deprivation, cancer cells adapt by expressing another lncRNA, gLINC, whose regulation is controlled by c-Myc. Once expressed, gLINC binds to the four downstream glycolytic enzymes, PGK1, ENO1, which interacts directly with PGAM1, PKM2 and LDHA. This promotes the formation of a metabolon that increases glycolytic flux, thereby balancing ATP production and preventing the cell from shunting metabolites towards the serine biosynthesis pathway. This ultimately serves to promote cancer cell survival. The figure was created with BioRender.

A recent study by Wang and colleagues^98^ revealed that the upregulation of aerobic glycolysis and the promotion of the Warburg effect can be facilitated by the interaction between the long non-coding RNA Highly Upregulated in Liver Cancer (lncRNA HULC) and two key glycolytic enzymes. HULC acts as a molecular scaffold, binding to LDHA and pyruvate kinase M2 (PKM2), thus converting them into RBPs. As a result, these enzymes become more susceptible to phosphorylation by the fibroblast growth factor receptor type 1 (FGFR1), which in turn leads to enhanced glycolytic activity and cell proliferation^98^ (Fig. 3a).

In addition to HULC, another important regulator of metabolic rewiring in cancer cells is the lncRNA glycoLINC (gLINC). This lncRNA is overexpressed in cancer cells in response to serine deprivation. The expression of gLINC is regulated by the c-Myc protein, which binds to a consensus sequence embedded in gLINC’s first exon. The elevated expression of gLINC in cancer cells under conditions of serine deprivation serves to maintain cellular viability. gLINC achieves this by acting as a scaffold, connecting four key enzymes of glycolysis. PGK1, ENO1, PKM2, and LDHA^99^ form a metabolon, a temporary structural-functional complex of metabolic enzymes, thereby enabling the glycolytic pathway to function^100^. The formation of this metabolon results in an increase in the glycolytic flux of cancer cells. This enhanced glycolytic activity serves to counteract the serine biosynthesis pathway (SBP). In the absence of this adaptation, serine deprivation would result in a reduction in ATP levels within the cells, thereby rendering them incapable of adapting to this metabolic stress. Consequently, gLINC plays a crucial role in enabling cancer cells to survive under challenging metabolic conditions, with the glycolytic enzymes PGK1, ENO1, PKM2, and LDHA functioning as RBPs (Fig. 3b).

In addition, METTL3, an RNA methyltransferase, has been described to facilitate tumour development by reducing the expression of *APC* (Adenomatous Polyposis Coli), a tumour suppressor gene. More precisely, METTL3 achieves this by adding the N6-methyladenosine (m6A) modification to the APC mRNA, which enhances its binding to the m6A “reader” protein YTHDF2. This interaction leads to the degradation of APC mRNA, which in turn leads to a reduction in APC protein levels and the abnormal activation of the Wnt/β-catenin signalling pathway. As a result, this activation causes a shift towards a more glycolytic state and carcinogenesis in mice^101^.

Another RBP that regulates glycolysis by altering PKM2 mRNA expression is DDX39B, which is involved in colorectal cancer (CRC) progression. DDX39B enhances the stability and nuclear translocation of PKM2, thereby activating it. The DDX39B-mediated PKM2 activation results in a metabolic shift towards aerobic glycolysis in CRC cells, promoting their proliferation, migration, and metastatic potential^102^.

### Examples of RNA-binding proteins as metabolic regulators in mitochondrial dysfunction

Ageing significantly impacts mitochondrial function, leading to structural abnormalities and dysfunction associated with various pathologies. One mechanism by which ageing promotes mitochondrial dysfunction, involves the RBP angiogenin (ANG), a member of the RNase A superfamily. During the ageing process, ANG undergoes dephosphorylation-induced translocation from the nucleus to the cytoplasm in glutamatergic neurons of the adult brain. This translocation enables ANG to interact with tRNAs, catalyzing the specific cleavage of nucleus-encoded tRNA^Glu^ into its transfer-RNA-derived small RNA, Glu-5’tsRNA-CTC. Subsequently, Glu-5’tsRNA-CTC interacts with leucyl-tRNA synthetase2 (LaRs2), an enzyme crucial for the ligation of mitochondrial tRNALeu (mt-tRNALeu) and leucine. The complex of Glu-5’tsRNA-CTC with LaRs2 translocates to the mitochondria, where LaRs2 is rendered incapable of aminoacylating mt-tRNA^Leu^, thereby impairing the translation of mitochondrial-encoded transcripts. This impairment leads to disrupted cristae organization, consequently affecting enzymes localized to this region, such as glutaminase, which is essential for glutamate biogenesis. Finally, the perturbation of glutamate homoeostasis results in synaptic disorganization and memory deficits^103^.

Another illustration of the manner in which ageing facilitates mitochondrial dysfunction via RBPs is the case of Pumilio 2 (PUM2), an RBP that typically functions as a translational repressor. PUM2 expression is elevated with age in multiple species, including worms, mice, and humans. It has been identified as a factor that impairs mitochondrial dynamics and mitophagy during the ageing process. PUM2 exerts its effects by inhibiting the expression of the mitochondrial fission factor (Mff) transcript, which is a major regulator of the mitochondrial fission machinery. This inhibition disrupts the fission between functional and dysfunctional mitochondria, consequently inhibiting the mitophagy of damaged mitochondria. Ultimately, this leads to an accumulation of dysfunctional mitochondria in the cell, contributing to age-related cellular decline^104^. Notably, PUM2 has been identified as a negative regulator of lifespan, suggesting that targeting this RBP could potentially offer therapeutic avenues for combating ageing-related mitochondrial dysfunction and extending healthspan^104^.

CPEB4 is an additional RBP that regulates the translation of mitochondrial-encoded transcripts, thereby influencing the mitochondrial proteome and, in turn, mitochondrial function. CPEB4 has been identified as a downregulated protein in numerous aged tissues, as well as in murine muscle stem cells. Proteomics analysis of these cells revealed a dual impairment of mitochondrial function and the mitochondrial proteome. CPEB4 is essential for the optimal functioning of MuSCs, as evidenced by the development of cellular senescence in MuSCs lacking this RBP. Conversely, the restoration of CPEB4 function has been shown to prevent cellular senescence and restore impaired mitochondrial metabolism^105^.

**Fig. 4:**
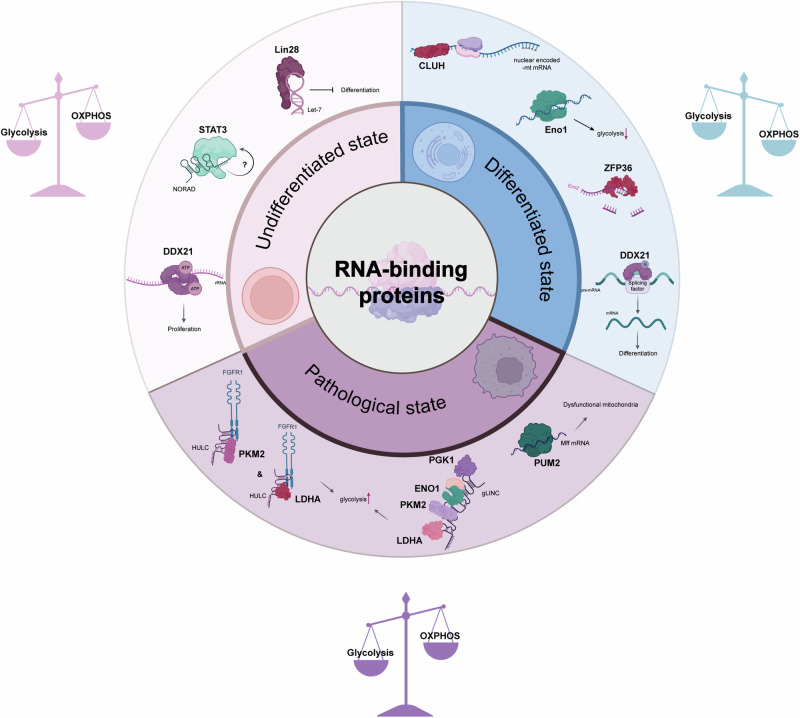
RNA-binding proteins as versatile metabolic regulators in health and disease. The circular diagram illustrates the three distinct states in which metabolic regulation is crucial, as outlined in this review. The three distinct states are those of undifferentiated stem cells, differentiated cells, and cells under pathological conditions. Each segment identifies specific RBPs that play key roles in regulating metabolism in the respective cell state. In the undifferentiated state, RBPs such as DDX21, LIN28, and STAT3 are responsible for maintaining pluripotency and promoting glycolytic metabolism. In differentiated cells, the prominent roles of CLUH, ZFP36, and an altered role of DDX21 facilitate the maintenance of high levels of OXPHOS in comparison to glycolysis. In the pathological state, particularly in cancer, RBPs such as PUM2, phosphorylated PKM2, and LDHA, along with the glycolytic metabolon formed by PGK1, ENO1, PKM2, and LDHA, indicate a shift in metabolic regulation contributing to disease progression. It is noteworthy that RBPs not only regulate RNA fate, but that RNA also influences the RBPs to which it binds. For instance, in the undifferentiated state, NORAD may affect STAT3 regulation, although the mechanism by which this occurs remains unknown. In differentiated cells, RNA exhibits an inhibitory effect on ENO1. Lastly, the lncRNAs HULC and gLINC modulate specific RBPs in the pathological state of cancer. The icons were created with BioRender.

In consideration of the examples described above, it is evident that RBPs play a pivotal role in orchestrating the metabolic changes that cells undergo during their transition from pluripotency through lineage specification and potentially into pathological states. These adaptive RBPs regulate gene expression at the post-transcriptional level, enabling rapid and precise responses to changes in the cellular environment during these processes. Fig. 4 provides an illustrative summary of some RBPs discussed in this review, with their roles in regulating metabolism.

## Future perspectives

The emerging field of RNA-binding proteins as regulators of cellular metabolism, particularly in stem cells, presents a promising avenue for future research. As our understanding of the complex interrelationship between RBPs and metabolic transitions deepens, several key areas require further investigation. A principal objective should be the creation of a comprehensive map of RBP-metabolic interactions. While considerable progress has been made in identifying specific RBPs involved in metabolic regulation, numerous interactions remain undiscovered. Future studies should employ high-throughput screening techniques to identify novel RBPs involved in metabolic regulation and develop more sensitive methods for detecting transient or weak RBP-RNA interactions that may play crucial roles in metabolic transitions. Furthermore, investigating the temporal dynamics of RBP-RNA interactions during different stages of stem cell differentiation and metabolic reprogramming will provide valuable insights into the regulatory mechanisms at play.

A deeper understanding of the precise mechanisms by which RBPs influence metabolic transitions is crucial for advancement of this field of study. It is of particular importance to ascertain how RBPs regulate the expression, stability, and localization of metabolic enzymes and metabolite transporters. The role of RBPs in regulating non-coding RNAs involved in metabolic transitions is a topic that warrants further investigation. Moreover, an investigation into the potential for crosstalk between RBPs and other regulatory factors, such as transcription factors and epigenetic modifiers, in orchestrating metabolic transitions would facilitate a more comprehensive understanding of the regulatory networks governing stem cell metabolism.

Several limitations currently hinder our ability to fully understand the role of RBPs in metabolic transitions. Functional redundancy among RBPs makes it challenging to delineate their individual contributions. The development of more sophisticated gene editing techniques and combinatorial knockout approaches may prove an effective means of addressing this issue. The context-dependent effects of RBPs on metabolism, which can vary depending on cell type and physiological state, necessitate more comprehensive characterization in future studies. Furthermore, technical challenges, such as the lack of spatial and temporal resolution in current methods for studying RBP-RNA interactions in vivo, must also be addressed. The development of enhanced in vivo imaging techniques and single-cell analysis methods will be crucial for capturing the dynamic nature of metabolic transitions.

As our understanding of RBP-mediated metabolic regulation grows, so too does the potential for translational applications. Future research should investigate the potential of RBP-targeted therapies for metabolic disorders and stem cell-based regenerative medicine. Furthermore, the potential of utilizing RBPs as biomarkers for metabolic states in stem cells and other cell types also holds promise. Additionally, an investigation into the potential of manipulating RBP activity to enhance or direct stem cell differentiation for therapeutic purposes could open new avenues in regenerative medicine.

To gain a more holistic understanding of RBP-mediated metabolic regulation, future studies should integrate multiple omics approaches. The integration of transcriptomic, proteomic, and metabolomic data will facilitate the development of comprehensive models of RBP-mediated metabolic networks. The development of sophisticated computational tools to integrate and interpret the complex data generated by these multi-omics approaches will be essential for extracting meaningful insights from these large-scale studies.

In conclusion, the study of RNA-binding proteins as metabolic regulators in stem cells and disease represents a rapidly evolving field with immense potential. By addressing current limitations, leveraging emerging technologies, and pursuing integrative approaches, future research can unlock new insights into the fundamental mechanisms of cellular metabolism. These advances will not only enhance our understanding of stem cell biology but also pave the way for novel therapeutic strategies in regenerative medicine and metabolic disorders. As we continue to unravel the complex interplay between RBPs and metabolism, we stand on the brink of exciting discoveries that could revolutionize our approach to cellular reprogramming and metabolic control.

## Data Availability

No datasets were generated or analysed during the current study.
